# Supplementary material for: Knowledge and utilization of family planning and safe abortion services among married women of reproductive age in the Raute community of Nepal: A census-based cross-sectional study
Source: PLOS Glob Public Health. 2026 Sep 2;6(9):e0006580. doi: 10.1371/journal.pgph.0006580 (PMC13537688; doi:10.1371/journal.pgph.0006580)
Supplement: Supplementary file 1 [file pgph.0006580.s001.docx]

| S.N. | Questions | Responses |  |
| --- | --- | --- | --- |
| 1 | Age |  |  |
| 2 | Family Type | Nuclear  Joint  Extended |  |
| 3 | Number Of family Member |  |  |
| 4 | Residence | Rural  Urban |  |
| 5 | Marital Status | Married  Unmarried  Divorced  Separated |  |
| 7 | If married, Age at first marriage |  |  |
| 8 | Occupation | Unemployed  Business  Service  House maker  Other…(Specify) |  |
| 9 | Occupation of Husband | Unemployed  Business  Service  Other…..(Specify) |  |
| 10 | Educational Status | Literate  Illiterate  Primary level (1-8)  Secondary level (9-12)  Higher Secondary level |  |
| 11 | Average Monthly Family Income | ………….. |  |
| 12 | Main Source of income | Agriculture  Animal Husbandry  Business  Job  Other………. |  |
| 13 | Which type of health services did you prefer to use? | Modern Health Care Services  Traditional Health care Services |  |
| 14 | If you use traditional health services, from where did you get these services? | Dhami  Jhakri  Unani  Baidhya  Others… |  |
| 15 | Currently Using any Family Planning Devices? | Yes  No |  |
| 16 | If no, then why? | Lack of money  Difficult to access  Husband Avoidance  Cultural Barrier  Lack of interest on using family planning devices  Others……. |  |
| 17 | If yes, What Type of family planning did you use? | Condom  OCPs  Depo  Implant  IUCD  Vasectomy  Female sterilization  Other………. |  |
| 18 | From which duration of time did you use particular device of family planning? | Less than a month  Month  Year  More than a year |  |
| 19 | In your opinion, what are the advantage of using family planning devices? | Birth spacing  Protection from STI  For the better health of both child and mother  Others |  |
| 20 | Did you know about safe abortion? | Yes  No |  |
| 21 | Did you Know from Where to get abortion Services? | Health Post  Private Clinic  Medical  Hospital  Others………. |  |
| 22 | Have you ever received any type of abortion services? | Yes  No |  |
| 23 | If yes, what type of abortion services did you receive ? | Medical abortion  Surgical Abortion |  |
| 24 | Did You Know About The Safe Abortion Policy of Government of Nepal ? | Yes  No |  |
| 25 | If Yes, What Type Of Services included in Safe Abortion Policy ? | Available up to 12 weeks of gestation on request  Available Up to 18 weeks’ gestation on cases of Rape or incest  Available at any time if the pregnancy poses a danger to women’s life or physical or mental health or there is a fatal abnormality  Others………. |  |
