# Supplementary material for: Knowledge and utilization of family planning and safe abortion services among married women of reproductive age in the Raute community of Nepal: A census-based cross-sectional study
Source: PLOS Glob Public Health. 2026 Sep 2;6(9):e0006580. doi: 10.1371/journal.pgph.0006580 (PMC13537688; doi:10.1371/journal.pgph.0006580)
Supplement: Supplementary file 2 [file pgph.0006580.s002.docx]

STROBE Statement — Checklist of items that should be included in reports of ***cross-sectional studies***

*Manuscript: Knowledge and utilization of family planning and safe abortion services among married women of reproductive age in the Raute community of Nepal: a census-based cross-sectional study*

| **Section/Topic** | **Item No** | **Recommendation** | **Page No. / Location in Manuscript** |
| --- | --- | --- | --- |
| **TITLE AND ABSTRACT** | | | |
| **Title and abstract** | 1a | (a) Indicate the study’s design with a commonly used term in the title or the abstract | Title page, p.1 — “a census-based cross-sectional study” stated in the title. |
|  | 1b | (b) Provide in the abstract an informative and balanced summary of what was done and what was found | Abstract, p.2- 3 — Background, methods (census, n=192, SPSS), results (FP 53.6%, SA 8.3%, AOR), and conclusions are all summarised. |
| **INTRODUCTION** | | | |
| **Background / rationale** | 2 | Explain the scientific background and rationale for the investigation being reported | Introduction, p.4-6 — Global and national FP/SA burden, indigenous health disparities, Nepal context, Raute community description, and evidence gap justified. |
| **Objectives** | 3 | State specific objectives, including any prespecified hypotheses | Introduction (last paragraph), p.6. |
| **METHODS** | | | |
| **Study design** | 4 | Present key elements of study design early in the paper | Methods – Study design, p.7 — “Census-based cross-sectional descriptive and analytical study” stated at the opening of the Methods section. |
| **Setting** | 5 | Describe the setting, locations, and relevant dates, including periods of recruitment, exposure, follow-up, and data collection | Methods – Study design, p.7 — Parshuram Municipality, Dadeldhura district, Sudurpaschim Province, Nepal. |
| **Participants** | 6a | (a) Give the eligibility criteria, and the sources and methods of selection of participants | Methods – Study design, p.7 — All 192 married women of reproductive age (15–49 years) in Raute community. Census method; exclusions: unmarried women, women with mental disability. |
| **Variables** | 7 | Clearly define all outcomes, exposures, predictors, potential confounders, and effect modifiers. Give diagnostic criteria, if applicable | Study variables, p.8 — Dependent variables (current FP use; ever SA use) and independent variables (age, family type, residence, income, literacy, etc.) explicitly listed. |
| **Data sources / measurement** | 8 | For each variable of interest, give sources of data and details of methods of assessment (measurement). Describe comparability of assessment methods if there is more than one group | Methods – Data collection tool, p.7-8 Structured questionnaire (3 sections), face-to-face interview in local language, pre-tested on 10% (n≈19) of comparable Raute community; daily review and double-entry verification. |
| **Bias** | 9 | Describe any efforts to address potential sources of bias | Methods – Data collection, p.7-8 — Interviews in private settings to minimise social desirability bias. Pre-testing to ensure cultural appropriateness. Double-entry verification to minimise data entry errors. Limitations section acknowledges possible underreporting of SA. |
| **Study size** | 10 | Explain how the study size was arrived at | Methods – Study design, p.7 — Census design; total eligible population (n=192) enrolled. No formal sample size calculation performed; rationale provided (small fully accessible population, maximises power for low-frequency outcomes). |
| **Quantitative variables** | 11 | Explain how quantitative variables were handled in the analyses. If applicable, describe which groupings were chosen and why | Methods – Study variables & Statistical analysis, p.8 — Continuous variables (age, income, children, family members) categorised; cut-points described in Table 1 and Methods. Groupings follow prior literature and clinical relevance (e.g., age ≤30 vs >30 years). |
| **Statistical methods** | 12a | (a) Describe all statistical methods, including those used to control for confounding | Methods – Statistical analysis, p.8 — IBM SPSS v16; descriptive statistics, chi-square for bivariate analysis; binary logistic regression (multivariable) with COR and AOR (95% CI). Variables with p<0.20 plus theoretical covariates entered in regression. |
|  | 12b | (b) Describe any methods used to examine subgroups and interactions | Methods – Statistical analysis, p.8. Subgroup analyses not formally performed; bidirectional examination of FP–SA association noted as pre-planned analytical approach. |
|  | 12c | (c) Explain how missing data were addressed | Methods – Data collection, p.7 Census design with 100% enrolment; no missing data reported. Double-entry verification ensured completeness. |
|  | 12d | (d) If applicable, describe analytical methods taking account of sampling strategy | Methods – Study design, p.7 Census sampling; all eligible participants enrolled; no complex sampling weights required. |
|  | 12e | (e) Describe any sensitivity analyses | Not applicable. No sensitivity analyses were performed. The study enrolled the entire eligible population (census design), precluding sensitivity analyses for sampling assumptions. This is acknowledged in the Limitations. |
| **RESULTS** | | | |
| **Participants** | 13a | (a) Report numbers of individuals at each stage of study | Methods – Study design, p.7 . Total Raute population: 419; eligible married women 15–49: 192; all 192 enrolled and analysed (100% participation rate). |
|  | 13b | (b) Give reasons for non-participation at each stage | No non-participation; census achieved 100% enrolment. Exclusion criteria (unmarried; mental disability) reported. No loss to follow-up in cross-sectional study. |
|  | 13c | (c) Consider use of a flow diagram | Flow diagram not included |
| **Descriptive data** | 14a | (a) Give characteristics of study participants and information on exposures and potential confounders | Results – Table 1, p.9-10 Full socio-demographic and socioeconomic characteristics presented with n and %. |
|  | 14b | (b) Indicate number of participants with missing data for each variable of interest | No missing data reported (census, 100% response rate, double-entry verification). This should be explicitly stated in the manuscript. |
| **Outcome data** | 15 | Report numbers of outcome events or summary measures | Results – Table 2, p.11-12 — Current FP use: n=103 (53.6%); SA ever used: n=16 (8.3%). Full breakdown of methods, duration, source, and reasons provided. |
| **Main results** | 16a | (a) Give unadjusted estimates and, if applicable, confounder-adjusted estimates and their precision (95% CI). Make clear which confounders were adjusted for and why | Results – Tables 3 & 4, p.13.16 — COR and AOR with 95% CI reported for all variables. Final adjusted model covariates stated in table footnotes. Confounders selected based on p<0.20 in univariate and theoretical relevance. |
|  | 16b | (b) Report category boundaries when continuous variables were categorised | Results – Tables 1, 3 & 4 — Category cut-points explicitly shown (e.g., age ≤30 / >30; income ≤7,000 / 7,001–10,000 / 10,001–20,000 NPR; children 0–2 / 3–5 / 6–10). |
|  | 16c | (c) If relevant, consider translating estimates of relative risk into absolute risk for a meaningful time period | Not applicable |
| **Other analyses** | 17 | Report other analyses done—eg analyses of subgroups and interactions, and sensitivity analyses | Bidirectional FP-SA Analysis performed. No other analysis were carried out. p.14 |
| **DISCUSSION** | | | |
| **Key results** | 18 | Summarise key results with reference to study objectives | Discussion, p.16-18 — Opening paragraph summarises the two primary findings (FP 53.6%; SA 8.3%; method mix imbalance; inverse FP–SA association) with direct reference to study objectives. |
| **Limitations** | 19 | Discuss limitations of the study, taking into account sources of potential bias or imprecision. Discuss both direction and magnitude of any potential bias | Strengths and limitations, p.19 — Cross-sectional design (no causal inference), social desirability bias (SA underreporting, true rate may be higher), small number of SA events (n=16, wide CIs), single-municipality setting (generalisability). Direction of bias discussed. |
| **Interpretation** | 20 | Give a cautious overall interpretation of results considering objectives, limitations, multiplicity of analyses, results from similar studies, and other relevant evidence | Discussion, p.16-18 — Findings contextualised against NDHS 2022, Mru Bangladesh, West Bengal tribal studies, and Tharu Nepal data. Limitations acknowledged before conclusions. Inverse FP–SA association labelled as “analytically important” not causal. |
| **Generalisability** | 21 | Discuss the generalisability (external validity) of the study results | Discussion – Strengths and limitations, p.19 — “The study was conducted in a single municipality and may not fully generalise to Raute populations in other districts.” Census design and unique nomadic context noted. |
| **OTHER INFORMATION** | | | |
| **Funding** | 22 | Give the source of funding and the role of the funders for the present study and, if applicable, for the original study on which the present article is based | No funding source for this study. |

** Items marked with * require author action before submission. See Page No. column for details.*

Note: An Explanation and Elaboration article discusses each checklist item and gives methodological background and published examples of transparent reporting. The STROBE checklist is best used in conjunction with this article (freely available on the Web sites of PLoS Medicine at http://www.plosmedicine.org/, Annals of Internal Medicine at http://www.annals.org/, and Epidemiology at http://www.epidem.com/). Information on the STROBE Initiative is available at www.strobe-statement.org.
