# Supplementary material for: Knowledge and utilization of family planning and safe abortion services among married women of reproductive age in the Raute community of Nepal: A census-based cross-sectional study
Source: PLOS Glob Public Health. 2026 Sep 2;6(9):e0006580. doi: 10.1371/journal.pgph.0006580 (PMC13537688; doi:10.1371/journal.pgph.0006580)
Supplement: Supplementary file 3 [file pgph.0006580.s004.docx]

**Author Response to Reviewer**

**‘Knowledge and utilization of family planning and safe abortion services among married women of reproductive age in the Raute community of Nepal: a census-based cross-sectional study’**

**(PGPH-D-26-01814)**

Dear Reviewer,

We sincerely thank you for your valuable time and insightful comments on our manuscript titled **“Knowledge and utilization of family planning and safe abortion services among married women of reproductive age in the Raute community of Nepal: a census-based cross-sectional study” (PGPH-D-26-01814)** submitted to PLOS Global Public Health.

We appreciate the constructive feedback, which has helped us significantly improve the quality, clarity, and rigor of our manuscript. We have carefully addressed all comments and revised the manuscript accordingly. Below, we provide a detailed, point-by-point response to each reviewer's comment. All changes have been clearly indicated in the revised manuscript with track changes and Highlights, and page/line numbers are provided for reference

| **Comments** | **Reviewer coment** | **Authors Response** | **Line No** |
| --- | --- | --- | --- |
|  | Data requests to a non-author institutional point of contact are required, along with details on long-term data storage and availability. | We have revised the Data Availability Statement to designate the Institutional Review Committee (IRC) of Manmohan Memorial Institute of Health Sciences as a durable, non-author point of contact for data access requests, in addition to the corresponding author. We have added the IRC's retention commitment for the study dataset. (email: info@mmihs.edu.np) | Page 22-23  Line (401-402 ) |
|  | Please review your reference list to ensure it is complete and correct, and confirm no cited papers have been retracted. | We have reviewed all 33 references cited in the manuscript and added three additional references in reference numbers 34, 35, and 36. | 26  Line No. ( 482-486) |
|  | If reviewer comments recommend citing specific previously published works, evaluate whether they should be cited. | Neither reviewer recommended specific additional citations; this item does not apply to our revision. | Not applicable |
| **Reviewer # 1** | | | |
| **R1.1** | The topic addressed in this article is important and highly relevant. However, the authors limit themselves to interpreting the data obtained from their quantitative study, neglecting the need to also critically examine it and enrich it with other theoretical contributions. Therefore, for the publication of this highly relevant and necessary text, it is essential to enrich theoretical dialogue precisely in the conclusions section, which is somewhat sparse and brief. Some elements that could help broaden this dialogue include: 1) the importance of comprehensive sex education, since access to family planning programs is not always sufficient to achieve gender equality and full access to human rights; 2) the right to reproductive health also involves understanding other rights, such as the right to a life free from violence and the connection to concepts like consent. Finally, although these issues are not the primary focus of the study, the conclusions would provide a suitable space to explore in greater depth certain elements that were only briefly mentioned, such as the male partner's commitment to family planning and the internalization of cultural norms as a barrier to family planning. Presenting the findings is important, however, the production of scientific knowledge also requires a critical discussion of their implications and limitations. | We thank the reviewer for this valuable and encouraging assessment, and we agree the Conclusions needed deeper theoretical engagement. We have added two new sentences to the Conclusions section of revised manuscript:   (1) On comprehensive sexuality education and right beyond access: service availability and reproductive autonomy are not synonymous: the concentration of contraceptive use in long-acting, provider-administered, female-directed methods, alongside near-total male non-participation, is consistent with a model in which women receive contraceptive commodities without a parallel foundation of comprehensive sexuality education, rights-based learning about the body, consent, and relationships that international guidance identifies as a necessary complement to service delivery, not a substitute for it. Without comprehensive sexuality education that addresses consent, bodily autonomy, and the right to a life free from violence, family planning gains carry the risk of treating symptoms rather than the underlying inequality of power that influences reproductive decision-making in this community. Access to contraception alone does not ensure reproductive autonomy. Family planning access on its own does not guarantee gender equity or the full realization of reproductive rights. Second, the right to reproductive health is inseparable from the right to a life free from violence and from the principle of free, informed, non-coerced decision-making. (2) On male partner commitment and internalized cultural norms: We now explicitly frame the near absence of male partner engagement in contraceptive decisions as both indicating that expanding access alone is unlikely to be sufficient. The near-complete absence of male-controlled methods and the concentration of contraceptive responsibility on women indicate that internalized cultural norms and low male partner commitment are structural barriers that cannot be captured by numerical uptake figures alone. Resolving these issues will require ongoing engagement with Raute men and community leadership, not just service expansion. Mobile reproductive health outreach, structured male-engagement strategies, legal literacy programs in the local language, and explicit recognition of nomadic indigenous communities in national reproductive health policy are indicated as priorities by these data; we present them as quantitatively-grounded starting points for action rather than as fully validated interventions, given that this cross-sectional, quantitative design cannot capture the cultural dynamics and social constraints that qualitative inquiry, as noted in our limitations, would be needed to explore | From line 347-374 |
| Reviewer #2 | | | |
| R2.1 | Although the data supports the conclusions, is desirable to qualify some arguments. Authors mention relevant concepts that match intercultural and intersectional frameworks, such as equity, gender equity, culturally appropriate; but this sensitivity is unsteady. Authors recognize that qualitative inquiry is needed to explore cultural dynamics and social constraints, as part of the limitations of the article; however, this doesn't qualify suggestions or arguments found on pages 2, 19 and 20, when referring what health authorities should do or when assessing "real progress" (p. 20). | We thank the reviewer for this important observation and agree our prescriptive language was not consistently qualified with the same caution we apply elsewhere regarding the need for qualitative inquiry. We have revised all three flagged locations:   (1) Abstract (p. 2) — "...are urgently required to improve reproductive health equity..." has been softened to Community-based mobile outreach tailored to nomadic movement patterns, legal literacy programs in the local language, and strategies to engage male partners are indicated as priorities by these quantitative findings; their design should be informed by qualitative, community-engaged research into the cultural and relational dynamics that a structured survey cannot capture.  , removing the unqualified imperative. (2) Programmatic and policy implications (p. 19) — every unqualified "should establish / is required / must be communicated / must be explicitly recognized" statement has been revised to hedge language ("could explore," "appears warranted," "could be communicated") and each recommendation is now explicitly tied to the need for consultation with Raute community leadership and further qualitative research, rather than presented as a direct conclusion from the quantitative findings alone. (3) Conclusions (p. 20) — "Family planning uptake at 53.6% reflects real progress" (an unqualified evaluative judgment) has been reworded to "...represents a substantially higher prevalence than might have been expected in this historically underserved community," which reports the descriptive finding without the normative claim the reviewer correctly identified as unsupported by our quantitative, cross-sectional design. | (1) (Page 3 , Lines 47-51). (2) (Page 19,20/ Lines 321-335) (3) )Page 21, Lines 337-342) |

We sincerely thank the editor and reviewers once again for their valuable feedback. We believe the revisions have significantly strengthened the manuscript and addressed all reviewer concerns. We hope it is now suitable for publication in PLOS Global Public Health.

Sincerely,
Mr. Manoj Joshi
(On behalf of all authors)
