# Supplementary material for: Knowledge and utilization of family planning and safe abortion services among married women of reproductive age in the Raute community of Nepal: A census-based cross-sectional study
Source: PLOS Glob Public Health. 2026 Sep 2;6(9):e0006580. doi: 10.1371/journal.pgph.0006580 (PMC13537688; doi:10.1371/journal.pgph.0006580)
Supplement: Peer review history

# Peer review: Decision Letter 0

**DOI:** 10.1371/journal.pgph.0006580.r001

**Authors:** María Medina Arellano

22 Jun 2026

PGPH-D-26-01814

Knowledge and utilization of family planning and safe abortion services among married women of reproductive age in the Raute community of Nepal: a census-based cross-sectional study

PLOS Global Public Health

Dear Dr. Joshi,

Thank you for submitting your manuscript to PLOS Global Public Health. After careful consideration, we feel that it has merit but does not fully meet PLOS Global Public Health’s publication criteria as it currently stands. Therefore, we invite you to submit a revised version of the manuscript that addresses the points raised during the review process.

Please submit your revised manuscript by Jul 22 2026 11:59PM. If you will need more time than this to complete your revisions, please reply to this message or contact the journal office at globalpubhealth@plos.org. When you're ready to submit your revision, log on to https://www.editorialmanager.com/pgph/ and select the 'Submissions Needing Revision' folder to locate your manuscript file.

Please include the following items when submitting your revised manuscript:

- A letter that responds to each point raised by the editor and reviewer(s). You should upload this letter as a separate file labeled 'Response to Reviewers'.
- A marked-up copy of your manuscript that highlights changes made to the original version. You should upload this as a separate file labeled 'Revised Manuscript with Track Changes'.
- An unmarked version of your revised paper without tracked changes. You should upload this as a separate file labeled 'Manuscript'.

Guidelines for resubmitting your figure files are available below the reviewer comments at the end of this letter.

As the corresponding author, your ORCID iD is verified in the submission system and will appear in the published article. PLOS supports the use of ORCID, and we encourage all coauthors to register for an ORCID iD and use it as well. Please encourage your coauthors to verify their ORCID iD within the submission system before final acceptance, as unverified ORCID iDs will not appear in the published article. *Only*  the individual author can complete the verification step; PLOS staff *cannot*  verify ORCID iDs on behalf of authors.

We look forward to receiving your revised manuscript.

Kind regards,

María De Jesús Medina Arellano, PhD

Academic Editor

PLOS Global Public Health

**Journal Requirements:**

1. In this instance it seems there may be acceptable restrictions in place that prevent the public sharing of your minimal data. However, in line with our goal of ensuring long-term data availability to all interested researchers, PLOS’ Data Policy states that authors cannot be the sole named individuals responsible for ensuring data access (http://journals.plos.org/plosone/s/data-availability#loc-acceptable-data-sharing-methods).

Data requests to a non-author institutional point of contact, such as a data access or ethics committee, helps guarantee long term stability and availability of data. Providing interested researchers with a durable point of contact ensures data will be accessible even if an author changes email addresses, institutions, or becomes unavailable to answer requests.

Before we proceed with your manuscript, please also provide non-author contact information (phone/email/hyperlink) for a data access committee, ethics committee, or other institutional body to which data requests may be sent. If no institutional body is available to respond to requests for your minimal data, please consider if there any institutional representatives who did not collaborate in the study, and are not listed as authors on the manuscript, who would be able to hold the data and respond to external requests for data access? If so, please provide their contact information (i.e., email address). Please also provide details on how you will ensure persistent or long-term data storage and availability.

If the reviewer comments include a recommendation to cite specific previously published works, please review and evaluate these publications to determine whether they are relevant and should be cited. There is no requirement to cite these works unless the editor has indicated otherwise.

Please review your reference list to ensure that it is complete and correct. If you have cited papers that have been retracted, please include the rationale for doing so in the manuscript text, or remove these references and replace them with relevant current references. Any changes to the reference list should be mentioned in the rebuttal letter that accompanies your revised manuscript. If you need to cite a retracted article, indicate the article’s retracted status in the References list and also include a citation and full reference for the retraction notice.

**Additional Editor Comments (if provided):**

Thanks for your contribution, we got 2 reviewers, one is asking for some reflections on some arguments that are needed in this discussion.

[Note: HTML markup is below. Please do not edit.]

Reviewers' comments:

Reviewer's Responses to Questions

**Comments to the Author**

1. Does this manuscript meet PLOS Global Public Health’s publication criteria?

Reviewer #1: Partly

Reviewer #2: Yes

**********

2. Has the statistical analysis been performed appropriately and rigorously?

Reviewer #1: Yes

Reviewer #2: I don't know

**********

3. Have the authors made all data underlying the findings in their manuscript fully available (please refer to the Data Availability Statement at the start of the manuscript PDF file)?

The PLOS Data policy

Reviewer #1: No

Reviewer #2: No

**********

4. Is the manuscript presented in an intelligible fashion and written in standard English?

Reviewer #1: Yes

Reviewer #2: Yes

**********

**Reviewer #1:**  The topic addressed in this article is important and, moreover, highly relevant. However, the authors limit themselves to interpreting the data obtained from their quantitative study, neglecting the need to also critically examine it and enrich it with other theoretical contributions. Therefore, for the publication of this highly relevant and necessary text, it is essential to enrich the theoretical dialogue precisely in the conclusions section, which is somewhat sparse and brief. Some elements that could help broaden this dialogue include: 1) the importance of comprehensive sex education, since access to family planning programs is not always sufficient to achieve gender equality and full access to human rights; 2) the right to reproductive health also involves understanding other rights, such as the right to a life free from violence and the connection to concepts like consent. Finally, although these issues are not the primary focus of the study, the conclusions would provide a suitable space to explore in greater depth certain elements that were only briefly mentioned, such as the male partner’s commitment to family planning and the internalization of cultural norms as a barrier to family planning. Presenting the findings is important, however, the production of scientific knowledge also requires a critical discution of their implications and limitations.

**Reviewer #2:**  Although the data supports the conclusions, is desirable to qualify some arguments. Authors mention relevant concepts that match intercultural and intersectional frameworks, such as equity, gender equity, culturally appropriate; but this sensitivity is unsteady. Authors recognize that qualitative inquiry is needed to explore cultural dynamics and social constraints, as part of the limitations of the article; however, this doesn't qualify suggestions or arguments found on pages 2, 19 and 20, when referring what health authorities should do or when assessing "real progress" (p. 20).

**********

what does this mean?). If published, this will include your full peer review and any attached files.

**Do you want your identity to be public for this peer review?**  If you choose “no”, your identity will remain anonymous but your review may still be made public.

For information about this choice, including consent withdrawal, please see our Privacy Policy

Reviewer #1: **Yes:**  Iris González Cortez

Reviewer #2: No

**********

[NOTE: If reviewer comments were submitted as an attachment file, they will be attached to this email and accessible via the submission site. Please log into your account, locate the manuscript record, and check for the action link "View Attachments". If this link does not appear, there are no attachment files.]

**Figure Resubmissions:**

# Author comment: Author response to Decision Letter 1

**DOI:** 10.1371/journal.pgph.0006580.r002

24 Jul 2026

Attachment Submitted filename: Response to reviewers.docx

# Editor report: Decision Letter 1

**DOI:** 10.1371/journal.pgph.0006580.r003

**Authors:** María Medina Arellano

4 Aug 2026

Knowledge and utilization of family planning and safe abortion services among married women of reproductive age in the Raute community of Nepal: a census-based cross-sectional study

PGPH-D-26-01814R1

Dear Mr Joshi,

We are pleased to inform you that your manuscript 'Knowledge and utilization of family planning and safe abortion services among married women of reproductive age in the Raute community of Nepal: a census-based cross-sectional study' has been provisionally accepted for publication in PLOS Global Public Health.

Before your manuscript can be formally accepted you will need to complete some formatting changes, which you will receive in a follow up email. A member of our team will be in touch with a set of requests.

Please note that your manuscript will not be scheduled for publication until you have made the required changes, so a swift response is appreciated.

IMPORTANT: The editorial review process is now complete. PLOS will only permit corrections to spelling, formatting or significant scientific errors from this point onwards. Requests for major changes, or any which affect the scientific understanding of your work, will cause delays to the publication date of your manuscript.

If your institution or institutions have a press office, please notify them about your upcoming paper to help maximize its impact. If they'll be preparing press materials, please inform our press team as soon as possible -- no later than 48 hours after receiving the formal acceptance. Your manuscript will remain under strict press embargo until 2 pm Eastern Time on the date of publication. For more information, please contact globalpubhealth@plos.org.

Thank you again for supporting Open Access publishing; we are looking forward to publishing your work in PLOS Global Public Health.

Best regards,

María De Jesús Medina Arellano, PhD

Academic Editor

PLOS Global Public Health

***********************************************************

Thank you for addressing all of the reviewers comments, it has brought the clarity they recommended. I believe this is an important contribution.

Reviewer Comments (if any, and for reference):
